# CT oral contrast and contemporary imaging pathways for suspected oesophageal perforation in the emergency department: a retrospective observational study

**DOI:** 10.1007/s10140-026-02532-9

**Published:** 2026-08-14

**Authors:** Samyak Shah, Adil Zia, Bruno Di Muzio

**Affiliations:** 1 Department of Radiology, Alfred Hospital, 55 Commercial Road, Melbourne, VIC 3004 Australia; 2 Department of Surgery, School of Clinical Sciences at Monash Health, Monash University, Melbourne, VIC Australia

**Keywords:** Oesophageal perforation, Oesophageal leak, Computed tomography, Oral contrast, Imaging pathways: Fluoroscopic oesophagography

## Abstract

**Purpose:**

To evaluate contemporary computed tomography (CT) imaging pathways for Emergency Department (ED) patients with suspected oesophageal perforation or leak, with a particular focus on oral contrast utilisation, radiologic findings and downstream investigations following the index CT examination.

**Methods:**

We performed a single-centre retrospective observational study of all adult ED patients who underwent CT imaging including the chest for suspected oesophageal perforation or leak between January 2015 and March 2026. Patients were identified through radiology database search. Demographics, clinical indications, oral contrast use, radiologic findings and investigations performed within five days of the index CT were recorded.

**Results:**

A total of 107 patients were included, with six (5.6%) confirmed oesophageal perforations. Oral contrast was administered in 62 (57.9%) CT examinations. Overall, 45 patients (42.1%) underwent downstream investigations, with similar rates regardless of oral contrast administration (43.5% vs. 40.0%). However, repeat CT was less frequent following oral contrast-enhanced CT (3.2% vs. 17.8%). Among patients with confirmed perforation, direct signs including oesophageal wall defect and extraluminal contrast leak were common (66.7% each), whilst indirect signs were also frequent present, including pneumomediastinum and pleural effusion (83.3% each). Pneumomediastinum was also frequently observed in patients without perforation.

**Conclusion:**

Rates of downstream investigation were similar regardless of oral contrast administration, however, repeat CT was less frequently performed following oral contrast-enhanced examinations. These findings suggest that oral contrast may influence subsequent imaging pathways. Prospective studies are warranted to determine whether selective oral contrast administration can improve diagnostic confidence while reducing unnecessary CT examinations.

**Graphical abstract:**

**Figure:**
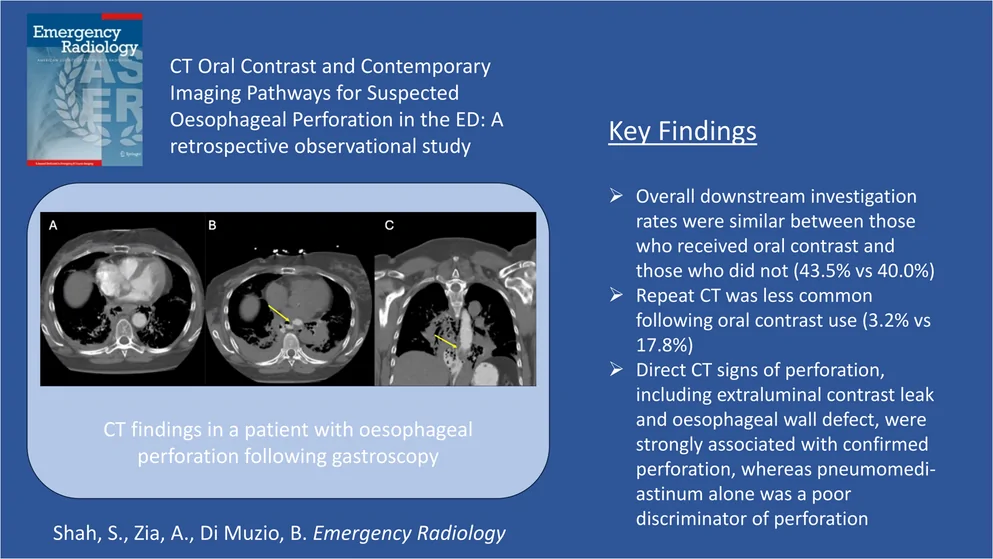

**Supplementary Information:**

The online version contains supplementary material available at https://doi.org/10.1007/s10140-026-02532-9.

## Introduction

Oesophageal perforation and leaks are rare but life-threatening conditions [1]. Perforation may arise from a variety of aetiologies, most commonly iatrogenic injury following endoscopic procedures, spontaneous rupture secondary to forceful retching (Boerhaave syndrome), and less frequently from foreign body ingestion, caustic ingestion, or trauma [2]. Oesophageal leaks occur due to failure of oesophagogastric anastomosis after surgical procedures such as oesophagectomy or fundoplication, which is performed for gastro-oesophageal reflux disease [3]. Clinical presentation is often non-specific and varies according to the site and cause of perforation, with symptoms including chest pain, vomiting, retching, and fever [2]. Delayed presentation may result in ongoing leakage of oesophageal contests into the mediastinum and necrosis of the oesophageal wall precipitating a rapid systemic inflammatory response, sepsis, and even cardiovascular collapse with multiorgan failure [4, 5]. In cases of oesophageal perforation, mortality is estimated to be 10–15% when treatment is initiated within 24 h, increasing up to 60% when management is delayed beyond 48 h [4]. In post-operative leaks after oesophagectomy, mortality ranges from 17 to 35% [6]. These outcomes underscore the importance of prompt diagnosis and timely intervention.

Historically, diagnostic evaluation of patients in the emergency department (ED) with suspected oesophageal perforation or leak relied on fluoroscopic oesophagography (FE) to demonstrate extraluminal contrast leakage [3, 4]. Water-soluble contrast agents are generally preferred initially because of the potential complications associated with mediastinal barium extravasation, although barium may improve sensitivity when the initial examination is negative [2, 7]. In practice, however, fluoroscopy carries several limitations in the ED, including patient cooperation, ability to swallow, appropriate positioning, and radiology staffing availability, particularly after hours [1]. Contemporary evidence increasingly supports contrast-enhanced computed tomography (CT), with or without oral contrast (CT oesophagography), as the primary imaging examination because of its high diagnostic accuracy and ability to identify alternative thoracic pathology [1, 8].

As a result, chest CT is frequently performed early in the diagnostic work-up of suspected oesophageal perforation in the ED [4]. CT offers rapid image acquisition, broad availability and the ability to assess for alternative or concomitant diagnoses such as aortic injury, pneumothorax, and peptic ulcer perforation. CT with oral contrast may demonstrate direct signs of perforation, including extraluminal contrast leak or oesophageal wall defect [3, 4, 9]. Even without the use of oral contrast, CT can reveal indirect signs of perforation such as pneumomediastinum, mediastinal fluid, pleural effusion and paraoesophageal gas [3, 10]. CT has been shown to be highly sensitive for detection and oral contrast administration may improve diagnostic confidence in suspected oesophageal perforation through visualisation of extraluminal leakage [11].

Although CT has become central to the evaluation of suspected oesophageal perforation, there is limited data describing contemporary ED imaging pathways, including the utilisation of oral contrast and the need for subsequent investigations [12]. This study aimed to evaluate CT imaging pathways in ED patients with suspected oesophageal perforation or leak, with particular emphasis on the utilisation of oral contrast CT, radiologic findings, and subsequent investigations following the index CT examination.

## Methods

### Study design and setting

This was a single-centre retrospective observational study conducted at a tertiary Level 1 trauma centre. The study was designed as a radiology-led audit of imaging pathways for suspected oesophageal perforation or leak in ED.

This study was conducted in accordance with STROBE guidelines for observational studies (Online Resource 1).

### Case identification

Patients were identified through a search of the Radiology Information System (RIS) using keywords “oesophageal perforation,” “esophageal perforation,” “leak,” “Boerhaave,” and “oesophageal injury.” Search results were manually reviewed to confirm relevance and to exclude cases where these terms were mentioned incidentally without clinical suspicion of oesophageal perforation.

### Inclusion and exclusion criteria

Adult patients (≥ 18 years) presenting to the ED between January 2015 and March 2026 who underwent CT imaging including the chest for suspected oesophageal perforation or leak were included.

**Fig. 1:**
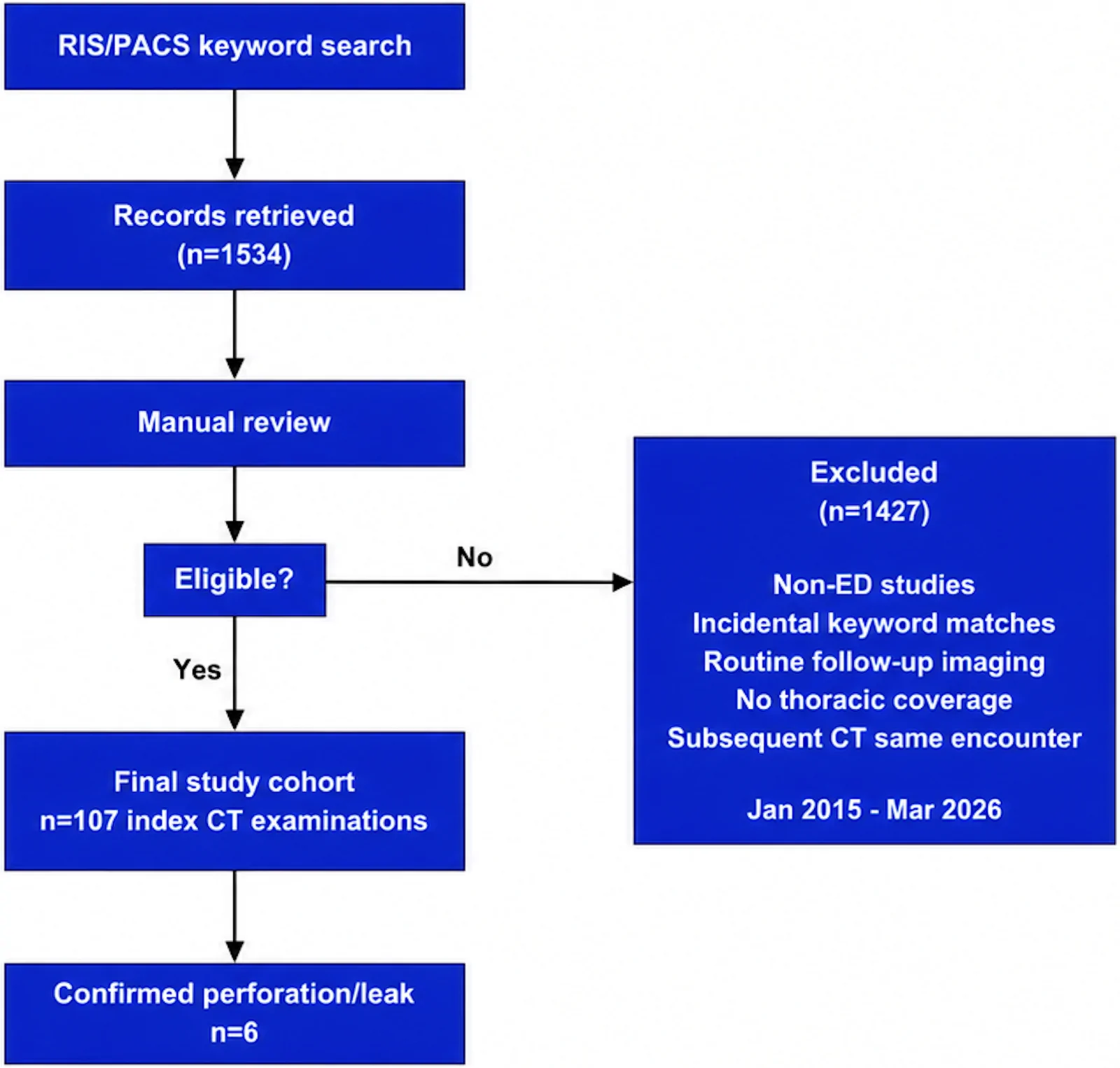
Flow diagram illustrating study cohort selection. Patients were identified through a RIS/PACS keyword search and underwent manual review against the study inclusion and exclusion criteria. A total of 107 index CT examinations were included in the final cohort, of which six demonstrated confirmed oesophageal perforation or leak

Studies were excluded if they were performed outside the ED, represented incidental keyword matches following manual review, comprised routine follow-up imaging of a known perforation or anastomotic leak, or did not include adequate thoracic coverage. Where multiple CT examinations were performed during the same emergency department encounter, only the index CT was included in the primary analysis. Subsequent CT examinations during the same encounter were excluded but recorded separately for descriptive analysis where relevant. The study selection process is summarised in Fig. 1.

### Data collection

Demographic and clinical data were obtained from the electronic medical record (EMR). The clinical indication for imaging was recorded from the request form and supplemented by EMR review where required. Indications were categorised as spontaneous (Boerhaave), post-procedural (e.g. gastroscopy or surgery), foreign body, chemical ingestion, non-specific presentations (e.g. chest pain), or specified as “other” which included traumatic presentations and food bolus impaction. For post-procedural cases, the type and timing of procedure were recorded separately.

CT-related data that was recorded included the scan type (e.g. CT Chest, CT Chest- Abdomen-Pelvis) and use of intravenous (IV) and/or oral contrast. In five cases, the oral contrast use was not clearly documented; these studies were subsequently reviewed by a radiologist with > 15 years’ experience to determine whether oral contrast had been administered.

Imaging findings, including both direct and indirect signs of perforation, were also recorded based on the report. Direct signs comprised of extraluminal contrast leak and oesophageal wall defect whilst indirect signs included pneumomediastinum, mediastinal fluid, paraoesophageal gas, and pleural effusion. For cases with equivocal findings on the initial report, the images were reviewed by an experienced radiologist to ensure accurate classification. The final radiology impression, as documented in the report, was categorised as perforation or leak present, indeterminate if a perforation or leak could not be excluded, and no perforation or leak. This radiologic classification was used as the primary outcome measure.

### Image acquisition

Most CT examinations in the ED were performed either without contrast or following administration of iodinated contrast, most commonly iohexol (Omnipaque 350), administered intravenously and/or dilute oral iodinated contrast were administered according to institutional protocol (prepared by diluting 30 mL of Omnipaque 350 in 250 mL of water).

When oral contrast was used, it was administered immediately prior to scanning, typically with an initial dose given in the upright position followed by an additional contrast in the supine position. Although prone imaging can further assist in evaluating anterior oesophageal abnormalities, it is not routinely performed in our institution.

Fluoroscopic oesophagography typically utilised a water-soluble contrast agent, most commonly Gastrograffin (meglumine diatrizoate), with Barium Sulfate used in only two cases.

### Reference standard and outcome verification

Given the radiology-led design of the study, the primary outcome was based on the radiologic impression on the index CT.

For cases in which the index CT was reported as positive or indeterminate for perforation, the reference standard for confirming clinically relevant oesophageal perforation was based on downstream investigations performed within 5 days of ED presentation. These were classified into primary downstream investigations, comprising repeat CT and fluoroscopic studies, and secondary investigations which included endoscopy and surgical intervention. In two cases, no downstream investigations were performed, the clinical course could not be established, and the outcome was deemed inconclusive.

For CT-negative cases, the absence of perforation or leak was determined based on downstream investigations where performed, together with clinical follow-up where available.

### Statistical analysis

Data was extracted and analysed using Microsoft Excel (Microsoft, USA). Descriptive statistics were reported as counts, percentages, and mean ± standard deviation or median (interquartile range) as appropriate.

Pre-test probability was derived from the clinical indication and categorised as:

- High, where perforation or leak was explicitly suspected or in spontaneous/post-procedural presentations.
- Moderate, where indirect features (e.g. pneumomediastinum) were the primary concern.
- Low, in all other cases.

Given the small number of confirmed perforations, no formal inferential statistical testing was performed, and analyses were considered descriptive.

This study was approved by the Alfred Health Human Research Ethics Committee (Project No. [433/26]). The requirement for informed consent was waived due to the retrospective nature of the study.

## Results

### Cohort description

A total of 107 patients presenting to ED with suspected oesophageal perforation or leak underwent CT imaging including the chest during the study period and were included in the analysis. Confirmed oesophageal perforation was identified in 6 patients (5.6%), while 99 patients (92.5%) had no perforation or leak. Two cases were deemed inconclusive: one patient died prior to further investigation and the other was discharged against medical advice and lost to follow-up.

Patient demographics and imaging characteristics are summarised in Table 1. The mean age of patients was 49.1 ± 20.6 years and 66.4% were male. The most common clinical indication was suspected spontaneous perforation (Boerhaave syndrome), accounting for 36.4% of cases (*n* = 39), followed by post-procedural presentations (25.2%, *n* = 29) and trauma-related presentations (15.9%, *n* = 17). Among post-procedural cases, gastroscopy with oesophageal dilatation was the most common preceding procedure (*n* = 8), followed by hiatus hernia repair (*n* = 5). CT Chest was the most frequently performed protocol (51.4%), followed by CT Chest-Abdomen-Pelvis (28%) and CT Chest + Abdomen (10.3%).

**Table 1:** Patient demographics, clinical indications and CT examination characteristics

Total patients, *n*	107
Male	71 (66.4%)
Female	36 (33.6%)
Age, mean ± SD	49.1 ± 20.6
Indication, n (%)	
Spontaneous (Boerhaave)	39 (36.4)
Post-procedural	29 (25.2)
Trauma	17 (15.9)
Chemical ingestion	10 (9.3)
Foreign body	5 (4.7)
Food bolus	2 (1.9)
Non-specific	5 (4.7)
CT Protocol, n (%)	
CT Chest	55 (51.4)
CT Chest-Abdomen-Pelvis	30 (28.0)
CT Chest + Abdomen	11 (10.3)
CT Pulmonary Angiogram	4 (3.7)
CT Thoracic Aorta	2 (1.9)
CT Pulmonary Angiogram + CT Abdomen-Pelvis	2 (1.9)
CT Aortogram	1 (0.9)
CT Neck + CT Chest-Abdomen-Pelvis	1 (0.9)
CT Neck + CT Chest	1 (0.9)
Contrast, n (%)	
Oral contrast only	20 (18.7)
IV contrast only	42 (39.3)
Oral + IV contrast	42 (39.3)
Neither oral nor IV contrast	3 (2.8)

### CT protocols and oral contrast utilisation

Oral contrast was administered in 62 of 107 examinations (57.9%), including 20 studies performed with oral contrast alone and 42 performed with both oral and intravenous contrast. Intravenous contrast without oral contrast was used in 42 examinations (39.3%), while three patients (2.8%) received neither oral nor intravenous contrast.

Oral contrast use varied according to pre-test probability and clinical indication. It was administered in 52.9% of high-probability presentations, all moderate-probability presentations, and 64.3% of low-probability presentations. Across the study period, utilisation varied between years but demonstrated an overall increase over time.

### Imaging findings

CT findings according to final outcome are summarised in Table 2. Among the six confirmed perforations, direct signs of oesophageal wall breach were common, with extraluminal contrast leak and oesophageal wall defect each identified in four cases (66.7%). Indirect signs were also frequently present, including pneumomediastinum and pleural effusion (83.3% each), while mediastinal fluid and paraoesophageal gas were identified in three cases (50.0%).

Among the 99 patients without perforation or leak, pneumomediastinum remained the most common finding (24.2%), followed by pleural effusion (11.1%). Mediastinal fluid and paraoesophageal gas were each identified in six patients. Notably, pneumomediastinum was observed frequently in both confirmed and non-perforation groups.

**Table 2:** CT findings according to final outcome

CT findings	Confirmed perforation/leak (*n* = 6)	No perforation/leak (*n* = 99)
Extraluminal contrast leak	4 (66.7%)	1 (1.0%)
Oesophageal wall defect	4 (66.7%)	0 (0%)
Pneumomediastinum	5 (83.3%)	24 (24.2%)
Pleural effusion	5 (83.3%)	11 (11.1%)
Mediastinal fluid	3 (50.0%)	6 (6.1%)
Para-oesophageal gas	3 (50.0%)	6 (6.1%)

### Downstream investigations

Downstream investigations following the index CT examination are summarised in Table 3. Forty-five patients (42.1%) underwent at least one downstream investigation within five days of ED presentation. Endoscopy was the most common investigation (32.7%), followed by fluoroscopic oesophagography (11.2%), repeat CT (9.3%) and surgery (6.5%).

Primary downstream imaging (repeat CT and/or fluoroscopic oesophagography) was performed in 17 patients (15.9%), while secondary investigations (endoscopy and/or surgery) were performed in 35 patients (32.7%). Seven patients underwent both primary and secondary investigations.

**Table 3:** Downstream Investigations following the index CT examination

Investigation	*n* (%)	Subgroup breakdown
Any downstream investigations	45 (42.1)	Imaging only (*n* = 10); interventional only (*n* = 28); both (*n* = 7)
Imaging investigations	17 (15.9)	CT only (*n* = 5); fluoroscopic oesophagography only (*n* = 7); both (*n* = 5)
Repeat CT	10 (9.3)	
Fluoroscopic oesophagography	12 (11.2)	
Interventional investigations	35 (32.7)	Endoscopy only (*n* = 28); surgery only (*n* = 0) endoscopy and surgery (*n* = 7)
Endoscopy	35 (32.7)	
Surgery	7 (6.5)	

### Oral contrast and downstream investigations

Overall rates of downstream investigation were similar between patients who did and did not receive oral contrast (43.5% vs. 40.0%; RR 1.09) (Table 4). Repeat CT was less frequent following oral contrast administration (3.2% vs. 17.8%; RR 0.18, RD − 14.6%). Rates of fluoroscopic oesophagography (12.9% vs. 8.9%), endoscopy (33.9% vs. 31.1%) and surgery (9.7% vs. 2.2%) were numerically higher among patients who received oral contrast.

**Table 4:** Downstream investigations according to oral contrast administration

Outcome	Oral contrast (*n* = 62)	No oral contrast (*n* = 45)	RR	RD
Any downstream investigation	27 (43.5)	18 (40.0)	1.09	+ 3.5%
Repeat CT	2 (3.2)	8 (17.8)	0.18	-14.6%
Fluoroscopic oesophagography	8 (12.9)	4 (8.9)	1.45	+ 4.0%
Endoscopy	21 (33.9)	14 (31.1)	1.09	+ 2.8%
Surgery	6 (9.7)	1 (2.2)	4.35	+ 7.5%

*RR* Relative risk, *RD* Risk difference. Comparisons are descriptive and no formal hypothesis testing was performed owing to the small number of outcome events

### Clinical indication and downstream investigations

Downstream imaging varied according to clinical indication (Online Resource 2). Repeat CT and fluoroscopic oesophagography were most frequently performed in the spontaneous perforation group (12.8% and 10.3%, respectively) and the post-procedural group (13.8% and 13.8%, respectively). Endoscopy rates were also higher in these two groups, performed in 41.0% of patients in the spontaneous perforation group and 31.0% of patients in the post-procedural group. In contrast, no patients in the chemical ingestion or foreign body groups underwent repeat CT or fluoroscopic oesophagography; endoscopy was performed in 50.0% of chemical ingestion cases and 20.0% of foreign body cases (Online Resource 2). 

**Fig. 2:**
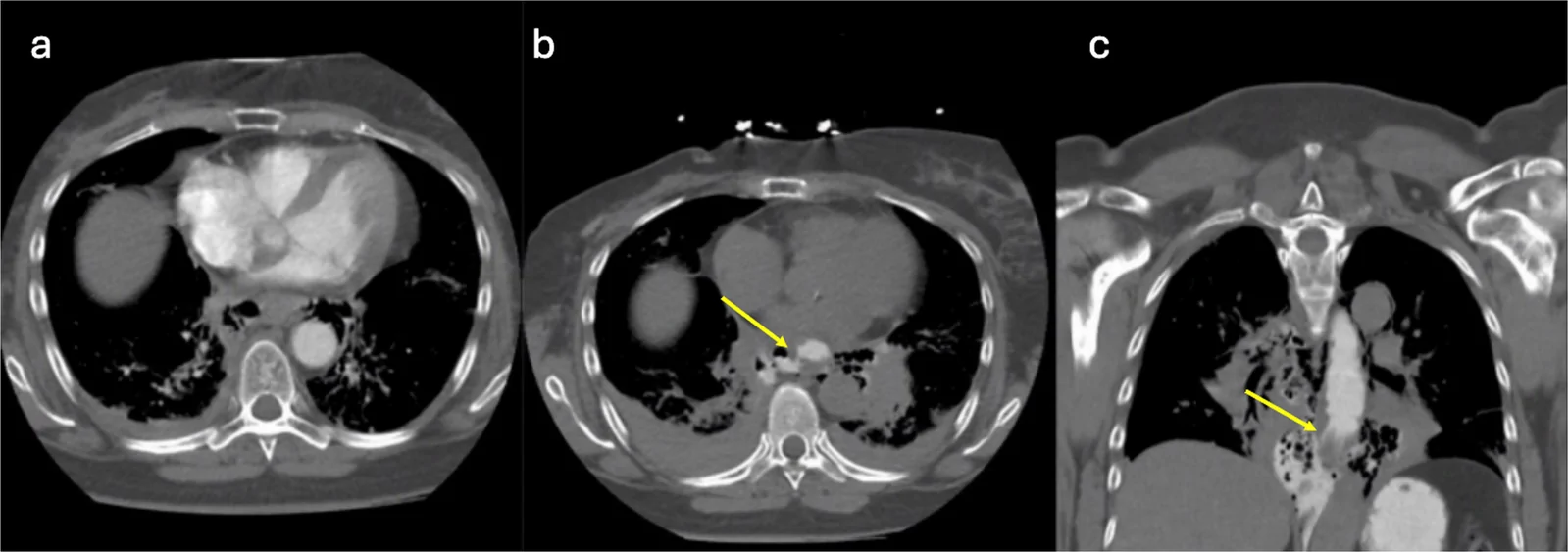
CT findings in a 55-year-old woman with oesophageal perforation following gastroscopy. The patient presented with severe abdominal pain, guarding and rigidity following gastroscopy. **a** Axial contrast-enhanced CT (portal venous phase) demonstrates extensive pneumomediastinum, highly suspicious for oesophageal perforation, although no definite site of perforation is identified. **b** Repeat CT following administration of oral contrast demonstrates focal contrast extravasation at the site of oesophageal perforation (arrow). **c** Coronal reformatted image from the oral contrast-enhanced CT better delineates the perforation site and associated contrast leakage (arrow)

The imaging findings of a representative case are displayed in Figure 2.

## Discussion

In this retrospective cohort of ED patients investigated for suspected oesophageal perforation or leak, confirmed perforation was uncommon, occurring in only 5.6% of cases. Oral contrast was administered in more than half of CT examinations, while downstream imaging was required in a minority of patients. Repeat CT was less frequently performed following oral contrast-enhanced examinations, although overall rates of downstream investigation were similar between oral contrast and non-oral contrast groups. Pneumomediastinum alone was a poor discriminator of perforation, whereas direct CT signs including oesophageal wall defect and contrast extravasation were strongly associated with confirmed perforation.

The low prevalence of confirmed perforation observed in our cohort is consistent with contemporary ED series, in which only 6–9% of patients investigated for suspected oesophageal perforation are ultimately diagnosed with the condition [1]. This reflects the role of CT in emergency practice as a rule-out investigation, where clinicians appropriately maintain a low threshold for imaging given the catastrophic consequences of delayed diagnosis and the often non-specific clinical presentation [13]. Consequently, ED cohorts differ substantially from surgical series, which are enriched for confirmed perforations through referral and selection bias.

Similar to previous studies, our cohort was predominantly male and most patients were investigated for suspected Boerhaave syndrome, post-procedural perforation or trauma, suggesting that it is representative of contemporary emergency practice [4, 12].

Oral contrast was administered in 57.9% of index CT examinations, closely aligning with the 53.4% utilisation reported by Wei et al. [1]. Our findings indicate that oral contrast remains commonly incorporated into contemporary emergency CT pathways despite limited evidence demonstrating substantial incremental diagnostic benefit over CT alone. Previous studies have shown that oral contrast provides only modest improvements in positive predictive value, while having little impact on the already high sensitivity and negative predictive value of CT [1]. Accordingly, contemporary guidelines support contrast-enhanced CT, with or without oral contrast, as the primary imaging examination for suspected oesophageal perforation [8]. The variation in oral contrast use observed in our cohort likely reflects differences in institutional practice, clinician preference and patient-specific factors rather than a consensus regarding its routine necessity.

In our institution, oral contrast is administered in dilute form (30 mL iohexol [Omnipaque 350] diluted in 250 mL water), providing adequate luminal opacification while minimising the aspiration-related risks associated with undiluted hyperosmolar water-soluble contrast agents [14].

Overall rates of downstream imaging were low, with repeat CT and/or fluoroscopic oesophagography performed in only 15.9% of patients. This supports previous studies demonstrating that CT alone appears sufficient to guide management in most patients presenting to the ED with suspected oesophageal perforation, with limited additional value from routine fluoroscopic oesophagography following a negative CT examination [1, 2, 15].

Although overall downstream investigations were similar irrespective of oral contrast administration, repeat CT was substantially less frequent following oral contrast-enhanced examinations. To our knowledge, this observation has not previously been described. One explanation is that oral contrast may improve diagnostic confidence by providing direct assessment of luminal integrity, thereby reducing the perceived need for further cross-sectional imaging. Alternatively, this finding may reflect differences in case mix and clinical decision-making. Patients who received oral contrast often had a higher pre-test probability or more complex clinical presentations, increasing the likelihood of targeted downstream investigations such as fluoroscopy, endoscopy or surgery rather than repeat CT.

Previous studies have compared the diagnostic performance of CT performed with and without oral contrast; however, no published studies have evaluated whether oral contrast influences downstream imaging or procedural investigations. Consequently, the optimal role of oral contrast within current ED imaging pathways remains uncertain. Our findings begin to address this gap by describing contemporary emergency department practice and identifying an association between oral contrast administration and reduced repeat CT utilisation.

These findings should be considered exploratory and hypothesis-generating. They suggest that oral contrast may influence subsequent imaging utilisation and support further prospective studies evaluating whether selective oral contrast administration can improve diagnostic confidence while reducing unnecessary repeat CT examinations.

The distribution of imaging findings across confirmed and non-confirmed perforation cases is broadly consistent with the existing literature. Pneumomediastinum was the most common CT finding among confirmed perforations, closely aligning with the findings reported by Awais et al. [11]. However, it was also the most common finding in cases without perforation, consistent with the observations of Evans et al., reinforcing its limited specificity as an isolated imaging sign, especially given the overlap with non-perforative causes such as the Macklin effect [4]. Specific CT features that have been reported to favour a non-oesophageal cause of pneumomediastinum include anterior compartment distribution, the presence of pulmonary interstitial emphysema, and the absence of subdiaphragmatic air or pleural effusion [1]. Importantly, Wu et al. And Wei et al. demonstrated that when pneumomediastinum is the sole abnormality on CT, the NPV for oesophageal perforation remains 100% [1, 16]. This suggests that isolated pneumomediastinum without other supporting features may not necessitate further imaging. In contrast, direct signs were considerably more specific; extraluminal contrast leak and oesophageal wall defect were each identified in 66.7% of confirmed perforations and in no cases without perforation, highlighting the greater specificity of direct CT signs when present.

All clinically confirmed perforations were identified on the index CT examination. While this observation is consistent with previous studies reporting high sensitivity of CT, the small number of confirmed perforations and non-uniform outcome verification preclude robust conclusions regarding diagnostic performance. Importantly, our findings should not be interpreted as suggesting that downstream investigations are unnecessary. CT cannot reliably exclude superficial mucosal injuries or contained mucosal tears without transmural perforation, and persistent clinical suspicion despite negative CT findings should therefore prompt endoscopic evaluation where clinically appropriate. Rather, fluoroscopic oesophagography, repeat CT, and endoscopy remain important complementary investigations in selected patients with equivocal CT findings, suspected post-operative leaks, or when additional anatomical characterisation is required to guide intervention [2].

Patterns of downstream investigation varied across indication categories. Repeat CT and fluoroscopy were the most common in spontaneous and post-procedural groups, likely reflecting the anatomical complexity of these cases and the need for detailed characterisation to inform operative management. This may also explain the higher rates of endoscopy observed within these groups; in cases with a positive index CT, endoscopy was used to confirm the diagnosis and assess the site and extent of injury prior to surgical intervention. Contrastingly, repeat CT and fluoroscopy were not performed in cases of chemical or foreign body ingestion, with endoscopic evaluation serving as both diagnostic and therapeutic.

In the post-operative setting, downstream imaging may be valuable in trying to detect oesophageal leaks. In patients who have undergone oesophagectomy, CT has been shown to have lower specificity for leak detection than in the native oesophagus, which may necessitate additional confirmatory imaging in this group [12]. Furthermore, the combined use of CT and fluoroscopic oesophagography for suspected post-operative leaks has been reported to achieve a sensitivity of 100%, suggesting a complementary role of fluoroscopy alongside CT in this context [17].

Based on our findings and the existing literature, CT with intravenous contrast supplemented by dilute oral iodinated contrast, when not contraindicated, should be considered as the preferred initial imaging examination in patients with suspected oesophageal perforation. This approach allows simultaneous assessment for direct signs of perforation, mediastinal and pleural complications, and alternative thoracic pathology, while potentially reducing the need for additional imaging in patients without evidence of contrast leak.

### Limitations

This study has several limitations. First, it was a retrospective single-centre study and therefore reflects local imaging practices, which may limit generalisability to other institutions. Second, despite an 11-year study period, confirmed oesophageal perforation was uncommon, with only six confirmed cases identified. Consequently, analyses of imaging findings, oral contrast utilisation and downstream investigations should be interpreted as exploratory and hypothesis-generating. Third, outcome verification was not uniform across the cohort. Patients with positive or indeterminate CT findings were more likely to undergo downstream investigations such as fluoroscopic oesophagography, endoscopy or surgery, whereas many patients with negative CT examinations were managed clinically without further confirmatory testing. This introduces the potential for verification bias and limits definitive assessment of diagnostic performance. Finally, oral contrast administration was determined by clinician preference and institutional practice rather than a standardised protocol. As a result, comparisons between oral contrast and non-oral contrast groups are susceptible to confounding by indication and should be interpreted cautiously.

## Conclusion

This study provides contemporary real-world data on CT imaging pathways for suspected oesophageal perforation in the emergency department. While overall downstream investigations were similar regardless of oral contrast administration, repeat CT was less frequently performed following oral contrast-enhanced examinations. These findings suggest that oral contrast may influence subsequent imaging pathways, although its optimal role remains uncertain. Prospective studies are warranted to determine whether selective oral contrast administration can improve diagnostic confidence while reducing unnecessary repeat CT examinations.

## Supplementary Information

Supplementary Material 1.

Supplementary Material 2.

## Data Availability

The data that support the findings of this study are not openly available due to reasons of sensitivity and are available from the corresponding author upon reasonable request. Data are located in controlled access data storage at Alfred Health.
