# Supplementary material for: CT oral contrast and contemporary imaging pathways for suspected oesophageal perforation in the emergency department: a retrospective observational study
Source: Emerg Radiol. 2026 Aug 14;33(5):1047–56. doi: 10.1007/s10140-026-02532-9 (PMC13633144; doi:10.1007/s10140-026-02532-9)
Supplement: Supplementary file 1 [file 10140_2026_2532_MOESM1_ESM.pdf]

Article title: CT oral contrast and contemporary imaging pathways for suspected oesophageal perforation in the emergency department: a retrospective observational study

Journal name: Emergency Radiology Journal

Author names: Samyak Shah[a], Adil Zia[a], Bruno Di Muzio[a,b]

Affiliations:

[a:] Department of Radiology, Alfred Hospital, 55 Commercial Road, Melbourne, Victoria, Australia 3004

[b:] Department of Surgery, School of Clinical Sciences at Monash Health, Monash University, Melbourne, Victoria, Australia

Corresponding author email: sam.shah@alfred.org.au

Online Resource 2: Table displaying downstream imaging based on clinical indication

Indication	Total	Repeat CT	Fluoroscopy	Endoscopy	Surgery
Spontaneous	39	5 (12.8)	4 (10.3)	16 (41.0)	3 (7.7)
Post-procedural	29	4 (13.8)	4 (13.8)	9 (31.0)	2 (6.9)
Trauma	17	1 (5.9)	2 (11.8)	2 (11.8)	2 (11.8)
Chemical ingestion	10	0	0	5 (50)	0
Foreign body	5	0	0	1 (20.0)	0
Non-specific	5	0	1 (20.0)	1 (20.0)	0
Food bolus	2	0	1 (50.0)	1 (50.0)	0

Online Resource 3: Table displaying oral contrast use across indications

Indication	Oral contrast	No oral contrast
Spontaneous (Boerhaave)	21 (53.8)	18 (46.2)
Post-procedural	16 (55.2)	13 (44.8)
Trauma	15 (88.2)	2 (11.8)
Chemical ingestion	4 (40.0)	6 (60.0)
Foreign body	2 (40.0)	3 (60.0)
Non-specific	3 (60.0)	2 (40.0)
Food bolus	1 (50.0)	1 (50.0)
